# Effect of Educational Tools on the use of Patient-Controlled Analgesia Devices

**DOI:** 10.4274/TJAR.2022.22988

**Published:** 2023-06-16

**Authors:** Olcayto Uysal, Serkan Karaman, Tuğba Karaman

**Affiliations:** 1 Clinic of Anaesthesiology and Reanimation, Tokat State Hospital, Tokat, Turkey; 2 Department of Anaesthesiology and Reanimation, Tokat Gaziosmanpaşa University Medical School Hospital, Tokat, Turkey

**Keywords:** General anaesthesia, hysterectomy, pain management, patient controlled analgesia, patient education, postoperative pain

## Abstract

**Objective:**

In the literature, there are confusing data about educational tools and device use. Therefore, it is not clear which method is superior to the other. The aim of this study was to evaluate the effects of educational tools on patient-controlled analgesia (PCA) usage in patients undergoing hysterectomy.

**Methods:**

Ninety-six patients undergoing hysterectomy were enrolled in the study. Patients were randomly assigned to a group (verbal, brochure, or video) consisting of 32 patients each using the closed envelope method. After operations, all patients were sent to the ward and evaluated with numerical rating scale score for pain at 15^th^ min., 2^nd^, 4^th^, 6^th^, 12^th^, 18^th^, 2^nd^, 4^th^, 6^th^, 12^th^, 18^th^, 24^th^ hours. Given dose, the number of button presses, presence of nausea and vomiting, and static and dynamic pain scores were recorded. During visits, patients who had a pain score ≥4 were administered paracetamol 1 g IV. Ondansetron 8 mg IV was injected into patients who had nausea and vomiting.

**Results:**

No significant differences were determined in resting and dynamic pain scores, number of button presses, and given doses between groups at 15^th^ min., 2^nd^, 4^th^, 6^th^, 12^th^, 18^th^, 24^t^h hours.

**Conclusion:**

In this study, education type did not affect PCA device use. We believe that whatever method the infrastructure of hospitals is suitable for, should be used for PCA device education.

**Main Points**

• Patient Controlled Analgesia devices play an important role in the management of postoperative analgesia. It is not known which method is superior among others in PCA device training. Each health center can provide device training in accordance with its own physical conditions.

## Introduction

Postoperative pain is known to be the cause of complications such as atelectasis, cardiopulmonary complications, and prolonged hospital stay, which impair the patient’s quality of life. Even acute pain can transform into chronic pain when treated improperly. Therefore, postoperative pain management is one of the cornerstones of the perioperative period.

Patient controlled analgesia (PCA) is widely used for postoperative pain management, and PCA devices allow patients to self-administer medications to relieve pain and are an effective method of postoperative pain control.^1^ It provides simple, fast and adequate pain relief without the need for a specialized anaesthesiologist.^2^ The intravenous (IV) method is commonly used, but some devices administer analgesics via oral, subcutaneous, epidural, or intrathecal ways.^1,3^ However, in order for this method works optimally, the device must be understood and used properly by the patient. For this reason, the patient should also consider how to use the device appropriately. However, surgical stress and postoperative pain can make the process of learning to use the device difficult.^4^ In some patients, the learning period may be time consuming.^4^ Disruptions related to this process may also lead to problems in postoperative pain control.

The education process can be performed verbally, using written sources such as brochures, or using visual education tools such as videos. There has been some disagreement about which educational tool is most effective in educating patients on the proper usage of PCA devices. Therefore, this study determines the effect of verbal, brochure, or video education on the use of PCA devices in patients undergoing a hysterectomy.

## Methods

After obtaining approval from the Tokat Gaziosmanpaşa University Clinical Research Ethical Board (17-KAEK-101) and registration to Clinical Trials (NCT03807960), this prospective randomized study was conducted with 96 [American Society of Anesthesiologists (ASA) I-II-III] patients scheduled for elective abdominal hysterectomy at Tokat Gaziosmanpaşa University Medical School Hospital between January 2018 and June 2019. The inclusion criteria of the patients were being aged 18-65 years, having ASA status of I-II-III, and being literate. None of the patients had PCA usage experience. Patients completed informed consent forms before participating.

Patients were randomly assigned to either the verbal group, brochure group, or the video group, each comprising 32 patients. For the patients participating in the verbal group, face-to-face meetings was performed on the day before surgery. The meetings were 15 min long and were carried out in quiet places to ensure an effective dialog between the educator and the patients. Patients were informed about how to use the PCA device (CADD-Legacy Smiths Medical Model 6300, St Paul, MN), and the educator addressed concerns such as overdose and fear of addiction. Patients in the brochure group were given an informative leaflet about how to use the PCA device, possible side effects, and concerns about overdose and fear of addiction on the day before the operation, which the patients were able to read until the time of operation. A short video consisting of general information written in the leaflet and on device usage was made for the video group, which patients watched for over and over one hour on a day before the surgery.

Patients in all three groups were instructed on the use of the 11-point numerical rating scale (NRS) system (0=no pain, 10=intolerable pain) for postoperative pain assessment at pre-operative visits. Patients were also informed of our aim to control postoperative pain using PCA devices; however, it was emphasized that pain may not completely disappear.

On the day of surgery, patients were monitored with electrocardiogram, non-invasive blood pressure, and pulse oximetry (SpO_2_). Anaesthesia was induced with fentanyl 1 µg kg^-1^ IV, propofol 2 mg kg^-1^ IV, and rocuronium 0.6 mg kg^-1^ IV. After denitrogenation with 60-80% O_2_ and 4 L min^-1^ fresh air supply, patients were intubated. Anaesthesia maintenance consisted of sevoflurane targeting mean alveolary concentration 1, along with an oxygen flow (50-50%), at a total gas flow of 4 liters. Before incision, morphine 0.1 mg kg^-1^ IV was administered (max. dose 8 mg) and 20 min before the end of the surgery paracetamol 1 g IV was administered. After extubation, PCA devices were implemented in the patients and the patients were taken to the recovery room where their follow-ups started. The timing of anaesthesia and surgery was also noted.

The drug solution was prepared by mixing 144 mL saline (0.09%) and 300 mg tramadol, giving a tramadol ratio of 2 mg mL^-1^. The device was programmed to inject tramadol 20 mg with lock-out interval of 10 min (max. 60 mg h^-1^) and maximum of three button presses per hour. The continuous infusion was not permitted. The first follow-up of the patients was performed at the 15^th^ minute in the recovery room. Patients were sent to the gynecology ward when the Aldrete score reached 10 points and were assessed at postoperative 2^nd^, 4^th^, 6^th^, 12^th^, 18^th^ and 24^th^ hours. Patients were asked whether they had pain, and pain scores at rest and during coughing were noted. Nausea and vomiting were also evaluated. Doses given, the number of button presses, and the presence of nausea and vomiting was recorded. Patient satisfaction with PCA education was evaluated with 10 point NRS (0: unsatisfied, 10: very satisfied) assessments were performed by anaesthesiologists.

During visits, patients who had a pain score ≥4 were administered paracetamol 1 g IV and patients with nausea and vomiting were administered ondansetron 8 mg IV. At the last visit, patients were asked whether they were satisfied with the PCA device, and discharge times were noted.

### Statistical Analysis

Data analysis was performed using the Statistical Package for Social Sciences (SPSS) 20.0 (SPSS Inc. Chicago, IL). Qualitative data were given as frequencies and percentages, whereas quantitative data were given as means and standard deviations. The Kolmogorov-Smirnov test was used to examine the data distributions, the Pearson chi-square test was performed to compare descriptive statistics, and the Kruskal-Wallis and Mood’s Median tests were used to compare groups that had non-normal distributions. Tukey’s HSD test was performed for post-hoc analysis; *P* < 0.05 was considered statistically significant.

Gülhaş et al.^5^ found a mean Visual Analog Scale (VAS) value of 3.9 ± 1.3 in cases in which PCA was used after hysterectomy. In our study, we predicted that education would improve the adaptation to the use of PCA and reduce the VAS value by 25%, and the sample size was calculated as 27 patients per group when the bilateral type I error was accepted as 0.05 and the power value was 0.80. Considering possible exclusions, at least 30 patients per group (total 90) were planned to be included in the study.

## Results

This study enrolled 96 patients who underwent hysterectomy. Patients were divided into three groups: verbal, brochure, and video, consisting of 32 patients each.

Table 1 shows the demographics of the patients. No significant differences were determined in the resting and dynamic pain scores between the groups at the 15^th^ min and at the 2^nd^, 4^th^, 6^th^, 12^th^, 18^th^ and 24^th^ hours (*P* > 0.05, *P* > 0.05 respectively). Table 2 shows the mean NRS scores of the patients with resting pain.

**Figure 1:**
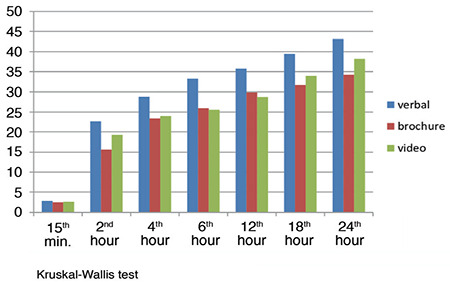
Number of Button Presses. PCA, patient-controlled analgesia.

**Figure 2:**
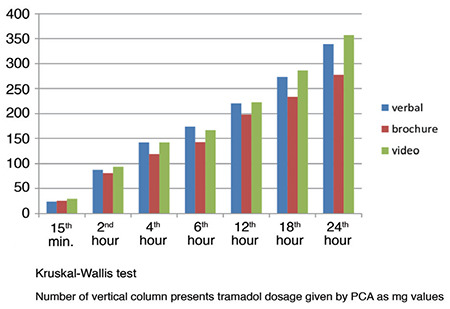
Given Doses by PCA.

Figures 1 and 2 show the number of button presses and given doses of the groups at the 24^th^ hour (*P* > 0.05, *P* > 0.05, respectively).

The number of patients requiring additional analgesics was 25 in verbal group, 19 in brochure group, and 15 in the video group. The presence of nausea was detected in 22 patients in the verbal group, 24 in the brochure group, and 19 in the video group. No significant difference was found among the groups in terms of the additional dose of analgesics and the presence of nausea (*P*=0.061, *P*=0.40, respectively).

Patients were asked whether they were satisfied with PCA education types. Mean values of verbal, brochure, and video groups were 9.28, 8.66, and 9.38, respectively (verbal min: 7 max: 10; brochure min: 4 max: 10; video min: 7 max: 10; assessed with 10 point NRS). Groups were compared for patient satisfaction, and no significant differences were found between the groups (*P*=0.175).

## Discussion

The findings of our study showed that no significant differences existed between the three education methods for PCA device use based on the patients’ resting and dynamic pain, number of button presses, and doses given. Additionally, no significant differences were found between the patient satisfaction scores.

PCA devices are widely used in postoperative settings, and it has been shown that these devices ensure a reliable way to achieve effective analgesia.^6^ However, as users need to be trained on how to use the device correctly, it is important to determine which type of training is most effective. Highly educated users will use the device more appropriately. This study determined the best education type for the correct use of a PCA device.

Patient education has developed considerably in recent years and can be performed in several ways, such as verbally, using written materials such as brochures, and using multimedia tools such as videos.^7^ Verbal education is one of the most commonly used methods in pre-operative education. Dealing with the patient directly and answering the patients questions make this method an effective educational tool. However, problems such as cultural differences between the patient and the educator and the time constraints of the healthcare professional providing the education should be considered.^8^ Written educational materials, such as brochures or leaflets, may solve the time constraints because the patient can read them at any time. Such materials should be written with short sentences and avoid technical or medical language; adding explanatory pictures to these materials increases intelligibility.^9^ Nevertheless, some patients may not understand these forms or may not even read them.^10^ Thus, video-assisted educational systems may be preferable because videos can be made more entertaining using animation. Videos are easy for patients watch and can be watched many times, as with brochures or leaflets. Additionally, the patient learning curve time may be decreased due to the live action style of content in videos. Nonetheless, these types of systems requires sophisticated equipment. All three methods can be used for patient education during the pre-operative period. Ascertaining the treatment steps, sharing treatment decisions with healthcare professionals, and enhancing recovery during the postoperative period are some well-known benefits of pre-operative education.^11^ Our study compared these three methods to determine which is most beneficial for PCA device usage.

The impact of education on PCA device usage is a challenging issue that has been investigated by many researchers. Although many studies claim that the type of education does not impact PCA usage, other studies show the superiority of certain methods. For instance, Chumbley et al.^12^ investigated parameters such as VAS scores, symptoms such as nausea and vomiting, and itching at the postoperative period to compare verbal and brochure methods on PCA device use. The findings revealed no significant difference between the methods. The results of their study are compatible with our results.

Some existing research also underlines the positive effect of structured education, which is formed by combining different types of education on PCA device usage. A study from South Korea performed with gynecological surgery patients gave videos and brochures about PCA device usage to the patients the day before surgery and compared the outcomes with patients receiving “in person” education. The findings showed that the structured education group was significantly more successful at pain control and experienced fewer side effects.^13^ Another study of orthopaedic patients showed that the structured education group used PCA devices more effectively than the routine education group; thus, their pain scores were significantly lower. The findings of this study underlined that when patients received only verbal education, they could misunderstand the knowledge, and when only written education was given, patients did not have the opportunity to ask questions. Researchers have emphasized that using both written and verbal methods together is the most effective way to perform patient education.^14^ Further, a study of cancer patients by Lovell et al.^15^ showed that using both videos and brochures was significantly more effective for patients compared to using these methods separately, as both methods reinforce the information given to the patient. Although we did not use any method in combination, analgesia and patient satisfaction in our groups met the desired level.

The impact of preoperative education on PCA device use has not only been researched among adult patients. In their study, Kotzer et al.^16^ questioned the effect of PCA device education in children. The patients were divided into two groups. The experimental group watched an eight-minute video about the purposes of pain treatment, drugs used in PCA devices, side effects of the drugs, etc., and the patients practiced using the device, had their questions answered, and were given a brochure. The routine education group was educated by different nurses each time. Instead of a standard verbal education, the nurses were free to elucidate their opinions about PCA device use, and patients were not allowed to practice using the PCA device; instead, the nurses explained how to use the device verbally. At the end of the study, the researchers determined that the total number of PCA demands and given doses in experimental and routine education groups were not significantly different.^16^ Furthermore, no statistically significant difference was found in the satisfaction of the patients’ families. The results of their study are consistent with our results. The researchers underlined that there was no statistically significant difference between the groups clinically, but it is important to inform the patients and caregivers about possible side effects, the drugs used in the device, and addiction development.^16^

Besides the pain parameters, no significant difference was observed between the scores recorded for nausea and vomiting. One of the well-known side effects of opioids is nausea and vomiting. In the literature, several articles have shown a relationship between tramadol usage and a high nausea and vomiting.^17,18^ In contrast, a study conducted by Ozalevli et al.^19^ in children undergoing tonsillectomy revealed that the tramadol-using group had a lower incidence of nausea and vomiting than the morphine-using group. None of our patients had treatment-resistant nausea or vomiting.

Respiratory depression, itching, and sedation are other side effects of using opioid-derived drugs with PCA devices. However, no studies have shown the superiority of one analgesic drug over another in terms of itching profile.^20^ Neither itching nor respiratory depression was seen in our patients. Furthermore, the basal infusion dose was not used in our study. Along with infusion doses making no contribution to pain management, increasing side effect incidence is a well-known impact of background infusion usage.^21^ Further, tramadol usage may cause less sedation compared to other opioids.^22^

Our study has several limitations. First, we did not evaluate the anxiety levels of patients. It is thought that preoperative education reduces patients’ anxiety levels.^9,23^ Second, no tests were performed to determine patients’ knowledge of the education materials. Instead of tests, we considered that the number of times the button was pressed would indicate the patient is to use the PCA device. Third, while the questions of patients in the verbal group were answered, patients in the brochure and video groups had no question-and-answer sessions.

The findings of our study showed that education type did not affect PCA device use. To provide adequate analgesia and ensure patient satisfaction with the PCA device, any method of education can be used according to the personnel and technical possibilities of hospitals.

## Figures and Tables

**Table 1:**
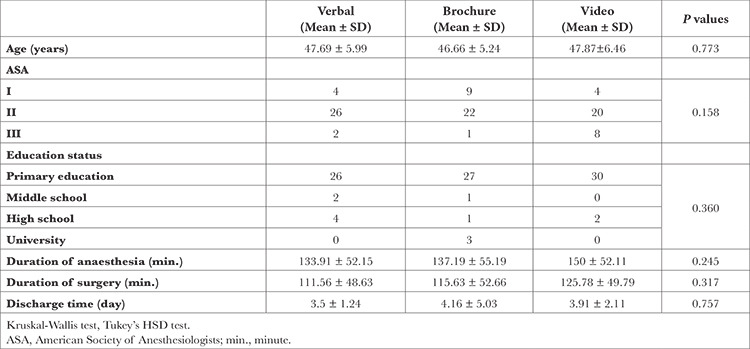
Patients Demographics

**Table 2:**
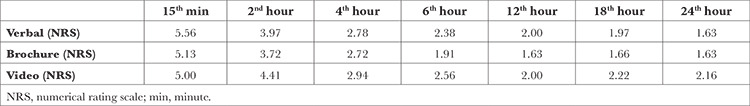
Mean Values of Pain Scores at First 24 Hour

## References

[1] Morlion B, Schäfer M, Betteridge N, Kalso E (2018). Non-invasive patient-controlled analgesia in the management of acute postoperative pain in the hospital setting. Curr Med Res Opin..

[2] Li JW, Ma YS, Xiao LK (2019). Postoperative Pain Management in Total Knee Arthroplasty. Orthop Surg..

[3] Nardi-Hiebl S, Eberhart LHJ, Gehling M, Koch T, Schlesinger T, Kranke P (2020). Quo Vadis PCA? A Review on Current Concepts, Economic Considerations, Patient-Related Aspects, and Future Development with respect to Patient-Controlled Analgesia. Anesthesiol Res Pract..

[4] Kissin I (2009). Patient-controlled-analgesia analgesimetry and its problems. Anesth Analg..

[5] Gülhaş N, Durmuş M, Yücel A, et al (2011). Total abdominal histerektomilerde intravenöz deksketoprofen trometamol, lornoksikam ve parasetamolün etkinliklerinin karşılaştırılması. Turk J Anaesthesiol Reanim.

[6] Dinges HC, Otto S, Stay DK, et al (2019). Side Effect Rates of Opioids in Equianalgesic Doses via Intravenous Patient-Controlled Analgesia: A Systematic Review and Network Meta-analysis. Anesth Analg..

[7] Rajala M, Kaakinen P, Fordell M, Kääriäinen M (2018). The Quality of Patient Education in Day Surgery by Adult Patients. J Perianesth Nurs..

[8] Oshodi TO (2007). The impact of preoperative education on postoperative pain. Part 1. Br J Nurs..

[9] Edwards PK, Mears SC, Lowry Barnes C (2017). Preoperative Education for Hip and Knee Replacement: Never Stop Learning. Curr Rev Musculoskelet Med..

[10] van Dijk JF, van Wijck AJ, Kappen TH, Peelen LM, Kalkman CJ, Schuurmans MJ (2015). The effect of a preoperative educational film on patients’ postoperative pain in relation to their request for opioids. Pain Manag Nurs..

[11] Giraudet-Le Quintrec JS, Coste J, Vastel L, et al (2003). Positive effect of patient education for hip surgery: a randomized trial. Clin Orthop Relat Res..

[12] Chumbley GM, Ward L, Hall GM, Salmon P (2004). Pre-operative information and patient-controlled analgesia: much ado about nothing. Anaesthesia..

[13] Hong SJ, Lee E (2012). Effects of a structured educational programme on patient-controlled analgesia (PCA) for gynaecological patients in South Korea. J Clin Nurs..

[14] Se H, HO CC, Zainah M, et al (2016). Structured education programme on patient controlled analgesia (PCA) for orthopaedic patients. Medicine and Health..

[15] Lovell MR, Forder PM, Stockler MR, et al (2010). A randomized controlled trial of a standardized educational intervention for patients with cancer pain. J Pain Symptom Manage..

[16] Kotzer AM, Coy J, LeClaire AD (1998). The effectiveness of a standardized educational program for children using patient-controlled analgesia. J Soc Pediatr Nurs..

[17] Harmer M, Davies KA (1998). The effect of education, assessment and a standardised prescription on postoperative pain management. The value of clinical audit in the establishment of acute pain services. Anaesthesia..

[18] Chen P, Chen F, Lei J, Zhou B (2020). Efficacy and safety of dexmedetomidine combined with tramadol for patient-controlled intravenous analgesia in Chinese surgical patients: A systematic review and meta-analysis. Medicine (Baltimore)..

[19] Ozalevli M, Unlügenç H, Tuncer U, Güneş Y, Ozcengiz D (2005). Comparison of morphine and tramadol by patient-controlled analgesia for postoperative analgesia after tonsillectomy in children. Paediatr Anaesth..

[20] Grass JA (2005). Patient-controlled analgesia. Anesth Analg.

[21] Jung H, Lee KH, Jeong Y, et al (2020). Effect of Fentanyl-Based Intravenous Patient-Controlled Analgesia with and without Basal Infusion on Postoperative Opioid Consumption and Opioid-Related Side Effects: A Retrospective Cohort Study. J Pain Res..

[22] Sağıroğlu G, Meydan B, İskender İ, et al (2011). Torakotomi sonrası analjezide, intravenöz tramadol ile hasta-kontrollü analjezi ve devamlı infüzyonun karşılaştırılması. Dicle Med J.

[23] Lemos MF, Lemos-Neto SV, Barrucand L, Verçosa N, Tibirica E (2019). A informação no pré‐operatório reduz a ansiedade pré-operatória em pacientes com câncer submetidos à cirurgia: utilidade do Inventário Beck de Ansiedade [Preoperative education reduces preoperative anxiety in cancer patients undergoing surgery: Usefulness of the self-reported Beck anxiety inventory]. Braz J Anesthesiol..

